# Rosemary Aqueous Extract as a Natural Alternative to Retinol for Skin Aging Intervention

**DOI:** 10.3390/ph19030378

**Published:** 2026-02-27

**Authors:** Ping Gao, Hong Zhang, Xuelan Gu

**Affiliations:** Unilever R&D Shanghai, 66 Linxin Road, Shanghai 200335, China

**Keywords:** *Rosmarinus officinalis*, retinol, anti-aging, inflammaging, collagen, dermal-epidermal junction, TRPV1

## Abstract

**Background/Objectives**: Retinoids are the gold standard for topical anti-aging treatments; however, their application is frequently limited by skin irritation and poor tolerability, particularly in sensitive or aged populations. Consequently, there is a growing demand for plant-based alternatives that offer comparable efficacy with an improved safety profile. The present study aims to explore the effects of rosemary aqueous extract (RE) on skin aging and its potential as a safe and effective alternative to retinol. **Methods**: Comparative RNA sequencing was employed to analyze the transcriptomic profiles of RE and retinol in human dermal fibroblasts (HDFs). Efficacy of collagen synthesis was evaluated using in vitro 2D and 3D skin models. As aging is associated with chronic inflammation, the responses of HDFs from young versus elderly donors under chronic IL-1β stimulation were compared, and a novel inflammaging model combining repetitive UVA irradiation with chronic cytokine (IL-1β and TNF-α) stimulation was utilized. Potential for neurogenic irritation was assessed by measuring transient receptor potential vanilloid subtype 1 (TRPV1) expression in SH-SY5Y neuronal cells. **Results**: RE was revealed to regulate gene expression in a pattern analogous to retinol, while also modulating distinct pathways related to wound healing and oxidative stress. RE not only enhanced collagen I synthesis but also protected against UVA-induced damage by preserving epidermal thickness, restoring the dermal-epidermal junction (DEJ), and reducing inflammation. Furthermore, RE demonstrated protective effects in the inflammaging model, effectively countering the synergistic damage caused by combined intrinsic and extrinsic stressors. Notably, RE downregulated TRPV1 expression in SH-SY5Y cells, suggesting a potential of reducing skin itching sensation. **Conclusions**: These findings position RE as a multifaceted anti-aging ingredient that not only represents a promising candidate for a retinol alternative, but also in the context of inflammaging and sensitive skin conditions, highlighting its potential impact on the future of anti-aging skincare.

## 1. Introduction

Skin aging is a multifactorial process resulting from cumulative interactions between skin and external environmental factors, primarily prolonged exposure to sunlight, combined with the intrinsic, genetically driven chronological aging. The retinoid family, which is chemically related to vitamin A, includes all-trans retinoic acid (ATRA, also known as tretinoin) and derivative compounds. They remain the most commonly used treatment for skin aging both clinically and cosmetically due to their collagen-promoting and pigmentation-reducing abilities [1,2]. However, the use of retinol is frequently limited by its poor tolerability, with the potential to cause skin irritation mediated through activation of the transient receptor potential vanilloid subtype 1 (TRPV1) receptor [3]. Stronger retinoids like tazarotene are considered unsuitable for geriatric populations due to consistently induced irritation, implying that aged skin is more susceptible compared to younger cohorts [4]. Another adverse effect is that retinoids, especially isotretinoin, have long been documented as teratogenic; thus, they are not recommended for use by pregnant women [5,6]. This has prompted a growing need in identifying natural, plant-based retinoid alternatives that can deliver comparable anti-aging benefits while minimizing adverse effects. Bakuchiol, extracted from Psoralea corylifolia seeds and often called “nature’s retinol”, has been shown by numerous studies to work on similar pathways as retinol to improve skin collagen production and cellular turnover, leading to enhanced texture and tone [7]. Additionally, it demonstrates potent antioxidant and anti-inflammatory properties and accelerates epidermal regeneration [8].

Rosemary (*Rosmarinus officinalis*) as an aromatic herb plant that offers culinary uses, is also known for its medicinal properties. Rosemary extract, derived from the leaves of the Rosmarinus officinalis plant, has emerged as a promising active ingredient for anti-aging skincare. It is composed of a plethora of bioactive molecules with great therapeutic potential, being particularly rich in antioxidants such as carnosic acid and rosmarinic acid [9], and has long been employed orally for its longevity-promoting and neuroprotective effects [10]. Moreover, rosemary extract has exhibited benefits for skin health, particularly regarding anti-aging and photoprotective effects in vitro [11,12,13]. The mechanisms underlying the anti-aging action of RE may include the promotion of collagen homeostasis [14], acceleration of elastic fiber formation [13] and prevention of UV-induced DNA damage [11,12]. Topically applied, rosemary extract was found to reduce the levels of inflammatory and wrinkle markers, thus providing photoprotection in rats under UVB exposure [15]. A recent study reported that a dietary supplement containing RE may offer significant improvements in facial skin quality after 12 weeks by counteracting glycation and glycation end products (AGEs) [16]. Importantly, in contrast with retinoids, rosemary extract is generally well-tolerated, with a much lower risk of irritation or adverse skin reactions when used in topical formulations or emulsions [17,18]. Reproductive and developmental toxicity is also not a concern with cosmetic use of rosemary-derived ingredients, which are mostly used at very low concentrations [18]. Phytexcell^TM^ rosemary is a cosmetic grade rosemary extract (RE), obtained from the leaves of *Rosmarinus officinalis*.

In the present study, we employed RNA-sequencing analysis to compare the gene expression profile and signaling pathways of RE and retinol, identifying RE as a potential natural retinol alternative. RE regulated gene expressions in a pattern similar to retinol but provided broader anti-aging benefits. In vitro experiments demonstrated that RE increased collagen production in both skin fibroblasts and 3D skin equivalents. Further, RE exhibited the capacity to protect against inflammaging in a new model combining stressors of chronic inflammation and repetitive UVA damage, which experimentally mimics the physiological skin inflammaging condition. Finally, RE decreased TRPV1 expression in SH-SY5Y cells, suggesting the ability to suppress neurogenic inflammation, thus reducing skin itch and irritation.

## 2. Results

### 2.1. Identification of RE as a Potential Natural Retinol Alternative by RNA-Sequencing Analysis

**Figure 1:**
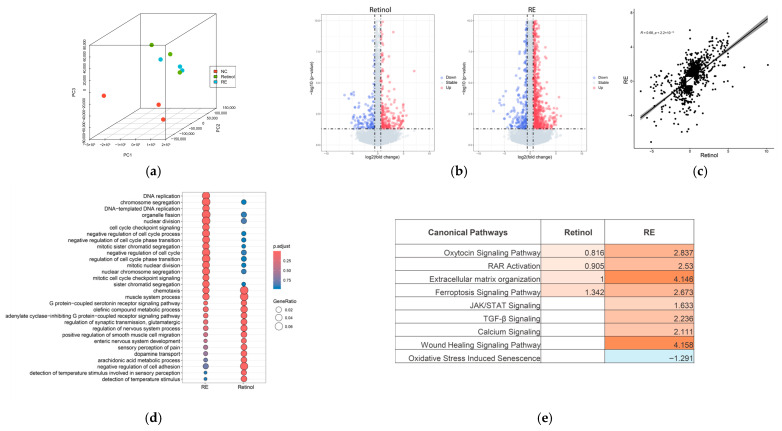
Comparative transcriptomic analysis of the effects of retinol and RE in HDFs. (**a**) PCA of whole transcriptome gene expression. (**b**) Volcano plot of differential gene expression under retinol and RE treatments. Red: up regulated genes (*p* < 0.05 and log2(fold change) > 0.58); blue: down regulated genes (*p* < 0.05 and log2(fold change) < −0.58). (**c**) Similarity of gene expression change between retinol and RE treatments based on union of differentially expressed genes. Line: regression line with a 95% confidence interval. (**d**) GO comparison analysis between retinol and RE treatments. (**e**) Pathway activities under retinol and RE treatments based on IPA pathway analysis. Color indicates the predicted pathway activity using IPA (z-score shown in the table).

First, we performed RNA-sequencing analysis and compared the RNA expression levels of the different sample groups. Principal component analysis (PCA) revealed that the transcriptomic profiles of retinol and RE-treated samples were distinct from the untreated control, while exhibiting similarity to each other (Figure 1a). Differential expression analysis revealed 1879 genes with *p*-value < 0.05 and fold change > 1.5 in the RE treatment group, whereas fewer genes (422) were identified under retinol treatment (Figure 1b). Fold changes in these combined genes were analyzed to predict the similarity in gene regulation between retinol and RE, resulting in a high similarity score of 0.68 (Figure 1c). Despite notable differences in the number of differentially expressed genes, GO enrichment analysis demonstrated that the top enriched GO terms were almost the same for both RE and retinol treatments, albeit with varying enriched gene ratios and *p*-values (Figure 1d). Additionally, IPA pathway analysis demonstrated the same result regarding functional similarity, showing that both retinol and RE activated RAR pathways and extracellular matrix (ECM) organization. Notably, RE potentially regulated additional pathways, such as TGF-β signaling, wound healing, and oxidative stress-induced senescence, indicating enhanced efficacy (Figure 1e).

### 2.2. Effect of RE on Collagen Production in Skin Fibroblasts

**Figure 2:**
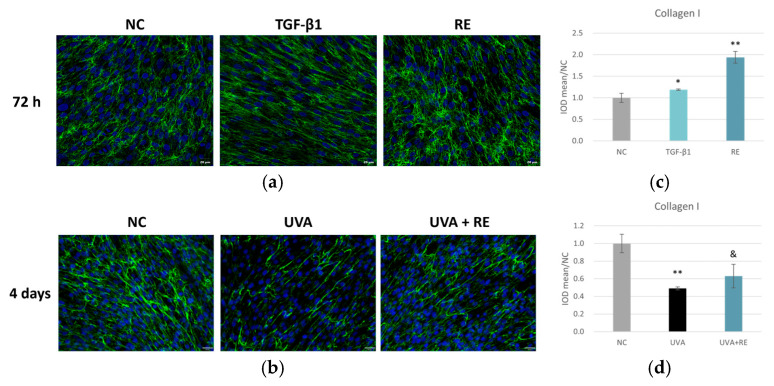
RE significantly enhanced collagen I synthesis in HDFs with or without UVA challenge. (**a**) Representative IF staining images for collagen I in HDFs treated with 100 ng/mL TGF-β1 or 0.1% RE for 72 h (scale bar = 20 µm). (**b**) Representative IF images for collagen I in HDFs exposed to 30 J/cm^2^ UVA for 4 days and treated immediately post-UVA with 0.1% RE (scale bar = 20 µm). Green staining represents collagen I. Blue staining (Hochest) represents nuclei. (**c**) and (**d**) present The Integrated Optical Density (IOD) of the signal of collagen I in (**a**) and (**b**), respectively. Data are presented as mean ± SD (*n* = 3). * *p* < 0.05, ** *p* < 0.01 versus NC; & *p* < 0.05 versus UVA.

We next investigated the effects of RE on collagen synthesis using in vitro HDF cultures. The application of TGF-β1, a known stimulator of collagen synthesis, increased collagen I levels to 119% of the negative control (NC), while the addition of 0.1% RE resulted in an even greater enhancement, reaching approximately 194% of the control (Figure 2a,c). This suggested that RE may potentiate collagen synthesis, surpassing the effect of TGF-β1. We also investigated the effects of RE on ECM remodeling in the context of UVA-induced skin damage. After four days of UVA irradiation, collagen I content was significantly reduced to approximately 49% of the control, whereas the RE-treated group demonstrated a notable recovery to 63% of the control (Figure 2b,d), indicating a protective or reparative effect against UVA-induced collagen I degradation. In summary, RE significantly enhanced collagen I synthesis in vitro and helped restore collagen levels after UVA-induced damage.

### 2.3. Dose-Dependent Effect of RE on the Production of Skin Structural Proteins and Inflammatory Cytokines in 3D Skin Models

**Figure 3:**
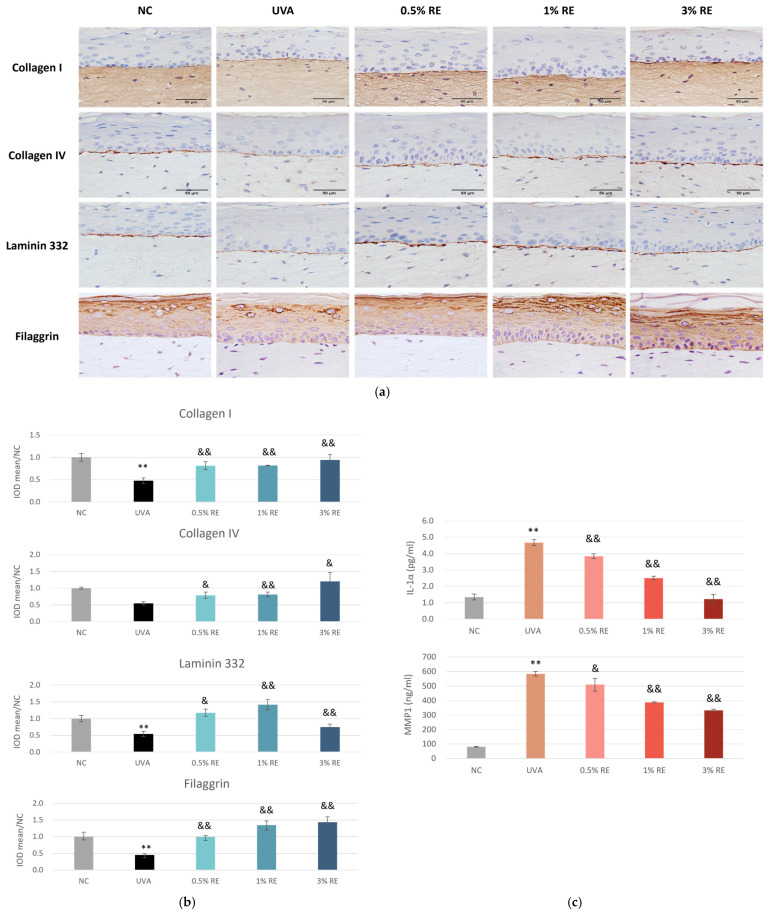
RE dose-dependently restored skin structural proteins and reduced inflammation in the 3D skin full thickness model under UVA exposure. (**a**) Representative IF staining images for collagen I, IV, laminin 332, and filaggrin in 3D full thickness skin models exposed to 35 J/cm^2^ UVA for 4 days and with RE applied topically at indicated concentrations at day 1 and 3 after UVA exposure (scale bar = 50 µm). (**b**) The IOD of the signal of collagen I, IV, laminin 332, and filaggrin, quantified using Image Pro Plus software 6.0 and graphed. (**c**) IL-1α and MMP1 levels measured in supernatants of 3D full thickness skin models treated with UVA and RE same as (**a**). Data are presented as mean ± SD (*n* = 3). ** *p* < 0.01 versus NC; & *p* < 0.05, && *p* < 0.01 versus UVA.

To further validate the photoprotective and regenerative potential of RE, immunohistochemical (IHC) staining was performed on 3D skin model sections following UVA irradiation. The expression of key structural markers, including collagen I, collagen IV, laminin 332 and filaggrin, was assessed at three concentrations of RE (0.5%, 1%, and 3%). The UVA group exhibited a marked decline of all four biomarkers compared to the NC group, signifying substantial photo-damage (Figure 3a). Collagen I expression in the dermis was significantly diminished following UVA exposure. However, treatment with RE demonstrated a clear dose-dependent recovery of collagen I levels. 3% RE treatment effectively restored collagen I levels to a state comparable to the NC (Figure 3a), suggesting a potent inhibitory effect on collagen degradation.

Additionally, the level of filaggrin—a critical indicator of epidermal barrier integrity—was severely compromised by UVA challenge, evidenced by sparse and weak staining in the stratum granulosum (Figure 3a). Application of RE as low as 0.5% significantly upregulated filaggrin expression, indicating that RE supported keratinocyte differentiation and reinforced the skin’s barrier against environmental stressors.

Moreover, the structural integrity of the dermal-epidermal junction (DEJ) was evaluated through the expression of collagen IV and laminin 332. In the NC group, these proteins formed a distinct, continuous linear band, whereas UVA challenge resulted in the thinning and partial loss of these proteins, highlighting the breakdown of the DEJ (Figure 3a). RE treatment mitigated collagen IV damage in a dose-dependent manner, resulting in a thicker and more defined linear staining pattern. For laminin 332, RE at all concentrations also effectively reversed the UVA-induced loss; but 3% showed lower efficacy, suggesting that increasing the dose to 3% might exceed the optimal threshold.

Inflammatory responses, together with the loss of skin structural proteins, may render the cutaneous tissue more prone to UV insults. The level of inflammatory cytokines was analyzed from the culture medium of 3D skin model sections following UVA irradiation. As shown in Figure 3c, skin models exposed to UVA produced significantly increased levels of IL-1α and matrix metalloproteinase 1 (MMP1), the enzyme responsible for collagen I degradation. Application of RE dose-dependently reduced the levels of both IL-1α and MMP1.

In summary, these findings demonstrated that RE provided significant photoprotection by preserving the dermal ECM, stabilizing the DEJ, maintaining epidermal barrier function, and reducing inflammation.

### 2.4. Effect of RE on Chronic Inflammation in Skin Fibroblasts Obtained from Both Young and Aged Donors

**Figure 4:**
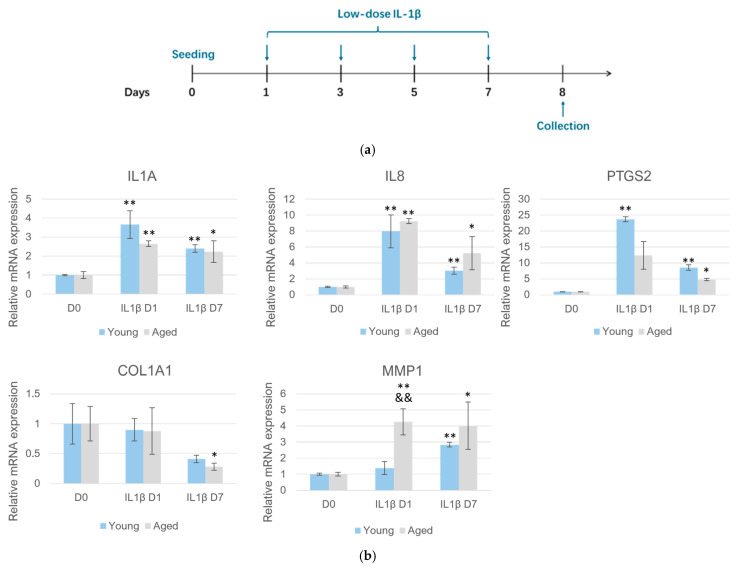
Responses of HDFs from young and aged donors under prolonged treatment of low-dose IL-1β. (**a**) Experimental schematic for examining the effect of low-dose, prolonged IL-1β (1 ng/mL) treatment on HDFs obtained from young (23 years old) and aged (56 years old) donors. (**b**) RT-qPCR analyses of HDFs from young and aged donors following treatment with IL-1β for 0, 1 or 7 days. D, day. * *p* < 0.05, ** *p* < 0.01 versus respective control at D0; && *p* < 0.01 versus young counterparts at the same timepoint.

In order to recapitulate the chronic, low-level inflammation during skin inflammaging, we examined the time-dependent response of HDFs to IL-1β at a relatively low concentration at 24 h and 7 days (Figure 4a). Aging is a dynamic process; examining skin cells from individuals of different ages can help reveal stage-specific responses and molecular targets. Therefore, we employed HDFs obtained from young (23 years old) and elderly (56 years old) donors. As shown in Figure 4b, the basal expression levels of pro-inflammatory genes such as *IL1A*, *PTGS2*, and *IL8* were found to be similar between cells from young and old donors, but acute stimulation of HDFs with IL-1β for 24 h resulted in marked increases in the expression of these genes. When HDFs were stimulated with IL-1β chronically for 7 days, the expression levels of inflammatory genes were still higher than non-stimulated cells but maintained at a lower level compared to those in the acute phase. This trend was similar in both young and aged HDFs.

However, the reaction pattern of genes of ECM homeostasis was different. *MMP1* expression was evidently lower in young HDFs compared to aged HDFs after 24 h of IL-1β stimulation. By day 7, *MMP1* level in aged cells steadily increased, but remained similar to that seen at 24 h, suggesting aged cells displayed a faster response of *MMP1* transcription activation to inflammatory signals. In both young and aged cells, the expression of *COL1A1*, which encodes the α1 chain of type I collagen, was not significantly affected following IL-1β exposure until D7, suggesting the negative impact on dermal collagen by chronic inflammation was a delayed, cumulative process (Figure 4b).

**Figure 5:**
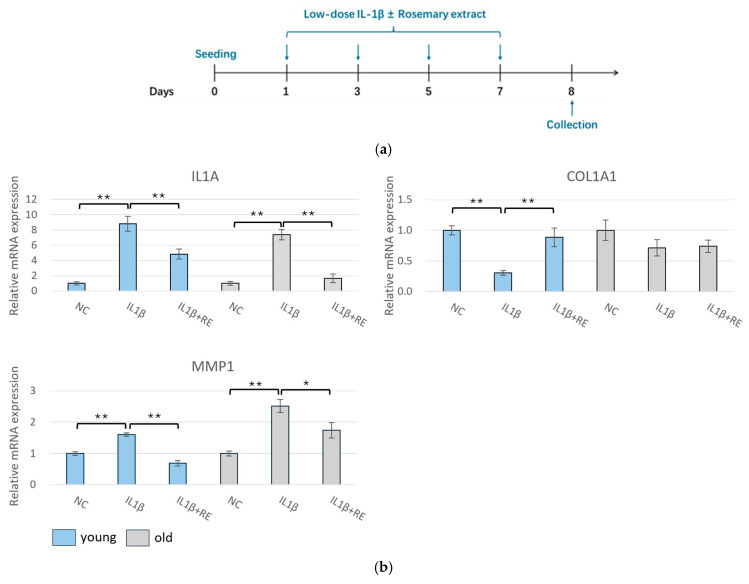
RE attenuated chronic inflammation and partially restored ECM homeostasis in HDFs from young and aged donors. (**a**) Experimental schematic for examining the effect of RE on HDFs obtained from young (23 years old) and aged (56 years old) donors under chronic IL-1β stimulation. (**b**) RT-qPCR analyses of HDFs from young and aged donors following treatment with IL-1β (1 ng/mL) with or without 1% RE for 7 days. * *p* < 0.05, ** *p* < 0.01. Data are presented as mean ± SD (*n* = 3).

We next sought to determine whether the effect of RE varied among cells originating from donors of differing ages when exposed to chronic inflammatory conditions (Figure 5a). As illustrated in Figure 5b, the treatment of RE restored the gene expression levels of *COL1A1* reduced by prolonged IL-1β treatment and inhibited the levels of *MMP1* and *IL1A* induced by IL-1β in young HDFs. Although the trend of gene changes was the same, no significant differences in *COL1A1* levels were observed in aged HDFs. These results may indicate that although RE could effectively suppress chronic inflammation, the ability of restoring ECM homeostasis disrupted by chronic inflammation appeared limited in aged cells.

### 2.5. Potential of RE to Protect Against Inflammaging in a Novel Model Combining Intrinsic and Extrinsic Stressors

**Figure 6:**
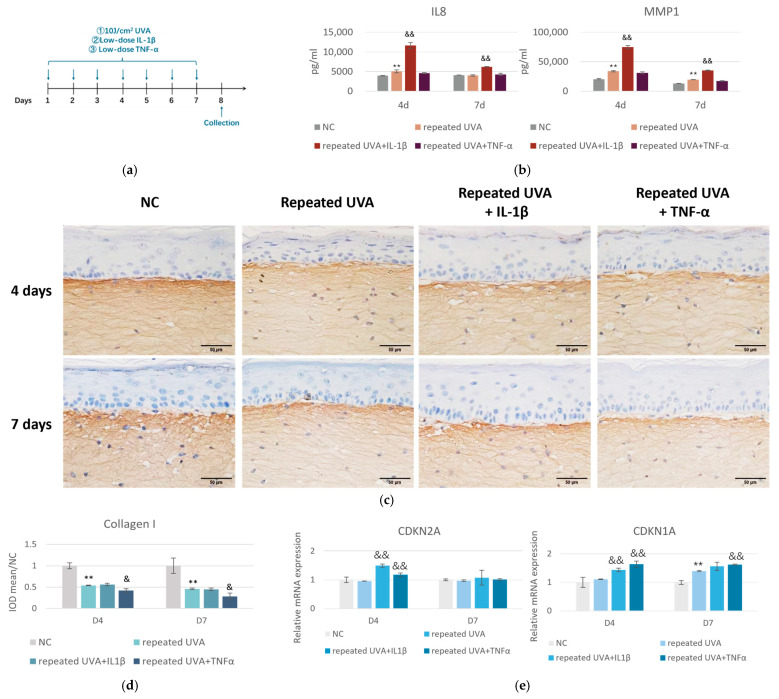
An in vitro inflammaging model revealed distinct, complementary roles for IL-1β and TNF-α under repeated UVA exposure in full thickness skin equivalents. (**a**) Experimental schematic of the 3D full thickness skin model designed to study inflammaging, which was subjected to dual stress comprising repeated UVA irradiation and exposure to low-dose inflammatory cytokines (IL-1β and TNF-α) over a 7-day period. (**b**) IL8 and MMP1 protein levels at D4 and D7 analyzed by ELISA, normalized to the NC. (**c**) Representative IHC images for collagen I and (**d**) quantification of the IOD of the signal at D4 and D7 (scale bar = 50 μm). (**e**) *CDKN2A* and *CDKN1A* expression at D4 and D7 analyzed by RT-qPCR. D, day. ** *p* < 0.01 versus respective NC; & *p* < 0.05, && *p* < 0.01 versus repeated UVA alone. Data are presented as mean ± SD (*n* = 3).

Given the anti-inflammatory properties of RE and the ECM disruption induced by inflammatory factors, it is imperative to further investigate the potential of RE to protect against skin inflammaging. Herein, we established an accelerated skin aging model to mimic in vivo skin inflammaging, utilizing the 3D full thickness skin equivalents. Skin aging is a multifactorial process driven by both intrinsic and extrinsic factors. In this model, we used chronic treatment of IL-1β and TNF-α at low concentrations as the chronological, intrinsic aging factor and low-dose, repeated UVA exposure as the environmental aging factor (Figure 6a). The data demonstrated that repeated UVA alone increased markers of inflammation (IL8) and ECM remodeling (MMP1) at the early stage (D4), but the induction was not persistent and returned to the baseline at D7. When IL-1β was introduced alongside repeated UVA, there was a pronounced synergistic effect, evidenced by a substantial increase in IL8 and MMP1 production compared to UVA alone at D4. Importantly, this effect sustained through D7 (Figure 6b), suggesting IL-1β amplified and maintained the inflammatory response induced by repeated, low-dose UVA exposure. By contrast, TNF-α did not enhance inflammation but exacerbated ECM remodeling, as illustrated by the IHC images of collagen I (Figure 6c,d). Importantly, the addition of either IL-1β or TNF-α further increased the mRNA expression of *CDKN2A* and *CDKN1A* at D4, which are senescence marker genes, but only TNF-α had a lasting effect on *CDKN1A* at D7 (Figure 6e). Together, these results supported our hypothesis that exposure to the combination of extrinsic (UVA) and intrinsic (age-related cytokines) factors accelerated the skin’s progression toward a state resembling inflammaging.

**Figure 7:**
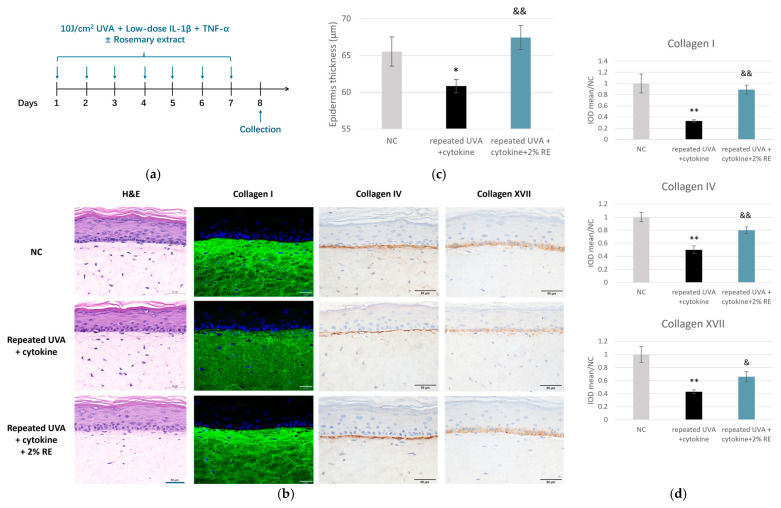
RE protected skin architecture and ECM integrity in an inflammaging 3D model. (**a**) Experimental schematic for examining the effect of RE on the 3D full thickness skin inflammaging model exposed to repeated UVA irradiation and combined cytokines (IL-1β and TNF-α) over a 7-day period. (**b**) Representative H&E images, and IF and IHC images for collagen I, IV, and XVII in 3D full thickness skin models (scale bar = 50 μm). (**c**) Quantification of epidermal living cell thickness and (**d**) the IOD of the signal for collagen I, IV, and XVII. * *p* < 0.05, ** *p* < 0.01 versus respective NC; & *p* < 0.05, && *p* < 0.01 versus repeated UVA alone. Data are presented as mean ± SD (*n* = 3).

Given the differences in the responses observed for the two cytokines, we treated skin models with both cytokines under the condition of repeated UVA exposure, so as to better recapitulate the complexity of inflammaging skin milieu (Figure 7a). Histological analysis via hematoxylin and eosin (H&E) staining revealed that the combined stressors employed herein induced significant structural deterioration, with a statistically significant reduction in epidermal thickness compared to the NC group. In contrast, treatment with 2% RE effectively preserved epidermal thickness, which was significantly higher than that of the inflammaging group and comparable to the levels in the NC (Figure 7b,c). Furthermore, the integrity of the ECM and the DEJ was evaluated through immunofluorescence (IF) and IHC staining analyses. In the inflammaging group, dermal structure was severely compromised, as evidenced by a sharp decline in collagen I density and significant disruption of the DEJ markers, collagen IV, and collagen XVII (Figure 7b). Quantitative analysis indicated that collagen I, IV, and XVII levels were reduced to approximately 35%, 50%, and 40% of the NC levels, respectively (Figure 7d). However, the application of 2% RE significantly attenuated these deleterious alterations. The RE-treated tissues showed robust recovery of dermal collagen density and improved integrity of the basement membrane, indicated by a thicker and more defined staining pattern (Figure 7b,d).

Collectively, these results confirmed the successful induction of the inflammaging process in this 3D model and demonstrated that RE protected against inflammaging by preserving key structural proteins of the dermis and DEJ, suggesting their further application in cosmetic formulations aimed at anti-inflammaging properties.

### 2.6. Effect of RE on TRPV1 Expression in SH-SY5Y Cells

**Figure 8:**
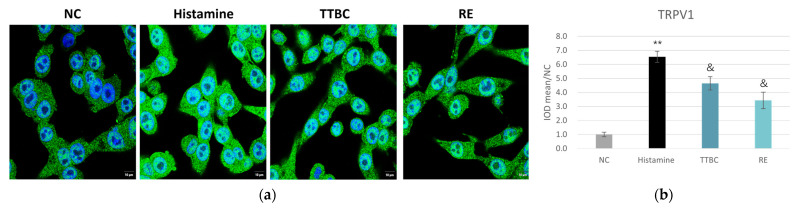
RE suppressed histamine-induced TRPV1 upregulation. (**a**) Representative IF staining images for TRPV1 in SH-SY5Y cells treated with 1% RE for 24 h (scale bar = 10 µm). Green staining represents TRPV1. Blue staining (Hochest) represents nuclei. (**b**) The IOD of the signal of TRPV1 in A). Data are presented as mean ± SD (*n* = 3). ** *p* < 0.01 versus NC; & *p* < 0.05 versus histamine.

Considering the irritation risk of retinoids, we set out to test the effect of RE on irritation by employing an in vitro model where the expression level of TRPV1 was evaluated in SH-SY5Y cells, a human neuronal cell line. This model serves as an indicator of neuronal hyper-responsiveness, which is a key element of stinging or burning skin phenotypes [19]. As shown in Figure 8, TRPV1 expression was significantly upregulated upon histamine stimulation. Antagonizing TRPV1 with trans-4-tert-butylcyclohexanol (TTBC) reduced histamine-evoked TRPV1 upregulation, while RE limited TRPV1 upregulation to an even greater extent, suggesting that RE was able to attenuate downstream calcium signaling and subsequent neurogenic inflammation, thereby prevent skin itching sensation in response to irritant stimuli.

## 3. Discussion

RE is a rich source of antioxidant and anti-inflammatory compounds, and has been recognized for addressing aging-related concerns, offering potential benefits for conditions like skin photoaging [15,20] and inflammatory and infectious skin pathologies [21]. However, there is currently a lack of studies directly comparing its efficacy head-to-head with retinoids, the most commonly used treatment for skin aging. In the present study, we provided a comprehensive analysis of RE as a natural retinol alternative for skin aging intervention for the first time, integrating approaches of computational, molecular, and cellular biology. Utilizing RNA sequencing for a comparative analysis, we found that RE modulated gene expression patterns that were analogous to those induced by retinol. Both compounds were found to converge on critical pathways regulating collagen synthesis, cellular turnover, and antioxidant defense, but RE displayed a broader regulatory scope encompassing pathways implicated in inflammation, wound healing, ferroptosis, and senescence (Figure 1e). These findings suggest that RE not only shares mechanistic overlap with retinol but also extends its activity profile, thereby potentially offering multifaceted skin protection and rejuvenation. Consistent with this interpretation, our in vitro data demonstrated that RE significantly enhanced collagen production in both 2D primary skin fibroblasts and 3D skin equivalents (Figure 2 and Figure 3), thereby positioning RE as a promising approach for skin aging and repair. Mechanistically, the promotion of collagen synthesis by RE could be attributed to its capacity to activate gene expression related with ECM organization, a finding supported by the RNA-sequencing results (Figure 1e). These observations align well with the previous literature reporting RE’s ability to stimulate elastic fiber formation and maintain collagen homeostasis [13,14].

The data in full thickness models showed that increasing concentrations of RE (0.5%, 1%, and 3%) led to a progressive, dose-dependent reduction in IL-1α and MMP1 levels (Figure 3), suggesting that RE is effective in suppressing skin inflammation and preventing ECM degradation. Consistently, in HDFs subjected to prolonged IL-1β treatment, RE exhibited robust anti-inflammatory efficacy by significantly attenuating the expression of common pro-inflammatory mediators (*IL1A*) and the tissue-degrading enzyme *MMP1* (Figure 5). Nonetheless, we observed that for the same gene, HDFs collected from young and elderly donors displayed different reaction patterns. For example, HDFs from the elderly donor expressed higher levels of *MMP1* than those from the young donor in response to chronic IL-1β stimulation (Figure 4). RE treatment significantly suppressed the induction of *MMP1* in cells from the young donor, whereas in cells from the elderly donor, the suppressive efficacy on *MMP1* was markedly attenuated (Figure 5). This discrepancy might reflect a decreased resilience in HDFs from the elderly donor to cope with stress under chronic inflammatory conditions, which aligned with prior research characterizing aged skin as inherently more fragile [4]. Taken together, the findings further substantiate the potential of RE as a natural alternative to traditional retinoids, given the latter’s limitation of causing irritation, and provide insight that different strategies of RE application may be needed for consumers of varying age groups. For older individuals, formulations with less irritative active ingredients would be more advantageous, with RE paired with agents offering soothing or calming benefits. However, it should be acknowledged that these findings are preliminary given the inclusion of only one donor per group. We are in the process of validating the results with cells derived from additional donors and future investigation using a larger cohort is warranted.

This anti-inflammation capacity is likely mediated through RE’s potent antioxidant constituents [8,9]. We thus wondered if RE could offer enhanced resilience against environmental insults and superior protection against inflammaging. Inflammaging, characterized by chronic low-grade inflammation and cumulative damage from environmental stressors, is increasingly recognized as a critical driver of skin aging [22]. Skin inflammaging is influenced by both intrinsic processes, such as psychosocial stress, and unhealthy lifestyles, which often involve chronic inflammation and extrinsic factors such as UV exposure, pollution, and high-energy visible light, all of which can accelerate the aging process [23]. While most studies have extensively focused on the effects of individual factors, the combined effects of intrinsic and extrinsic factors have not been thoroughly explored. We employed a novel in vitro model in the present study that combined chronic stimulation of inflammatory cytokines and repetitive UVA exposure to mimic the complex milieu of inflammaging in the skin (Figure 6). IL-1β and TNF-α were used as representative intrinsic aging factors, not only because they are highly prominent cytokines in systemic aging with established aging-related roles [24,25,26], but also because emerging evidence indicate that they can mediate skin aging [27,28,29,30,31,32]. Our preliminary studies demonstrated that prolonged treatment of IL-1β and TNF-α for 7 days at a low concentration resulted in low-grade inflammation, in contrast to a more pronounced inflammatory response observed during the acute phase at 1 or 3 days. The combination with either cytokine with UVA resulted in a higher expression of senescence marker genes than UVA alone (Figure 6), suggesting that repeated UVA radiation in conjunction with intrinsic inflammatory factors accelerates the progression toward an inflammaging state. However, IL-1β and TNF-α exhibited distinct actions in our skin models: IL-1β amplified the inflammatory response, while TNF-α exacerbated ECM degradation (Figure 6). Therefore, we examined the effects of RE under a combined challenge with IL-1β and TNF-α, in addition to repeated UVA. As shown in Figure 7, the combination induced a synergistic tissue deterioration in skin equivalents. A decrease in epidermal thickness was observed, accompanied by reduced levels of DEJ collagens (types IV and XVII) as well as dermal collagen (type I). In this aggressive environment, RE treatment still effectively prevented epidermis thinning and restored dermis integrity, which underpinned RE’s capacity to address not only photoaging and chronological aging but also the accelerated aging process driven by complex interplay of chronic inflammation and environmental stressors. However, skin inflammaging is a complicated process involving cell senescence, ECM remodeling, and the decline and dysregulation of cutaneous immune responses. Our simplified model could not fully recapitulate the intrinsic complexity. Additional work on the more sophisticated in vitro models for skin inflammaging, such as 3D models integrating immune cells (e.g., macrophages or Langerhans cells), are imperative to provide a more holistic and accurate understanding of the underlying mechanisms.

One of the principal clinical limitations of retinol-based therapies is their propensity to induce neurogenic inflammation and skin irritation, predominantly mediated through the activation of the TRPV1 receptor [3]. The current study revealed that RE significantly decreased TRPV1 expression in SH-SY5Y cells (Figure 8), suggesting another advantage RE holds over retinoids. Existing literature also corroborates the favorable safety profile of RE, noting its low risk of irritation and absence of reproductive toxicity when used in topical formulations [17,18]. By mitigating TRPV1-mediated neurogenic inflammation, RE could inhibit sensory hypersensitivity associated with burning, pruritus, and irritation, thus enhancing product tolerability. Although this has meaningful clinical implications, particularly for patient populations sensitive to conventional retinoids, it is important to note that TRPV1 protein expression should be considered as an indirect indicator of neurogenic irritation, rather than as definitive evidence of functional changes. Additional data such as calcium channel activation, neuronal activation readouts, and sensory responses are required to draw definitive conclusions regarding the efficacy of RE to modulate sensory sensitivity.

Despite the promising results above, the absence of separate clinical studies restricts the ability to extrapolate these findings to in vivo settings. Rigorous randomized controlled clinical trials are indispensable to validate both clinical efficacy and safety across diverse human cohorts. In addition, continued investigations into the precise molecular basis of RE’s modulation of neurogenic and inflammatory pathways will further elucidate its mechanism of action and inform the development of next-generation anti-aging therapeutics.

## 4. Materials and Methods

### 4.1. Cell Culture and Treatment

For the experiment of collagen I IF staining, HDFs provided by Guangdong Biocell Biotechnology (Dongguan, Guangdong, China) were plated in 6-well plates. When confluency reached 50%, cells were treated with 0.1% RE (Phytexcell^TM^ Rosemary, Croda International; INCI: ROSMARINUS OFFICINALIS (ROSEMARY) EXTRACT; Product Code: NA34518) or TGF-β1 (Peprotech, Cranbury, NJ, USA) at a dose of 100 ng/mL. Cells were continuously cultured for 72 h before collagen I IF staining. For UVA-challenged HDFs, all groups except the non-treated, NC group were irradiated with UVA (30 J/cm^2^) in phosphate buffer saline (PBS) followed by immediate medium refreshment with or without 0.1% RE after UVA irradiation. The plates were then cultured for 24 h before collagen I IF staining.

For the comparison of HDFs from donors of different ages, HDFs (Lifeline^®^ Cell Technology, Frederick, MD, USA) isolated from a healthy elderly donor (abdominal, age 56 years, male), and a young donor (abdominal, age 23 years, male) were cultured in fresh Dulbecco’s Modified Eagle Medium (DMEM, Gibco, Brooklyn, NY, USA) supplemented with 10% Fetal Bovine Serum (FBS, Gibco). The medium was refreshed every other day. Cells before passage 7 were plated in 6-well plates. When confluency reached 50%, cells were treated with IL-1β (Proteintech, Rosemont, IL, USA) at a dose of 1 ng/mL, with or without 1% RE in medium supplemented with 1% FBS. Cells were continuously cultured for 7 days before culture supernatant was collected for enzyme-linked immunosorbent assay (ELISA) analysis, and RNA was collected for real-time quantitative PCR (qPCR) analysis.

The human neuroblastoma cell line SH-SY5Y was provided by Guangdong Biocell Biotechnology. Cells were plated in 24-well plates. When confluency reached 50%, cells were treated with histamine (1 mM), TTBC (TCI, Shanghai, China, 15.6 μg/mL; CAS RN: 21862-63-5), or 1% RE and continuously cultured for 24 h before IF staining of TRPV1.

### 4.2. RNA-Sequencing Analysis

HDFs (FB220309, Guangdong Biocell Biotechnology, Dongguan, Guangdong, China) were seeded in a 6-well plate until the confluency reached 30%~50%. Then cells were treated by 10 μM retinol (Tauto Biotech, Shanghai, China; INCI: RETINOL) or 1% RE separately. After incubation for 48 h, cells were collected and sent to Berry genomics (Beijing, China) for RNA sequencing.

RNA concentration was measured using a NanoDrop 2000 (Thermo Fisher Scientific, Waltham, MA, USA) and RNA quality was determined using an Agilent 4200 (Agilent Technology, Santa Clara, CA, USA). RNA sequencing was conducted using an Illumina NovaSeq 6000 by Berry Genomics (Beijing, China).

Clean reads were mapped to the human genome (hg38) using Hisat2. Differential expression analysis was conducted using both edgeR and DEseq2. Differentially expressed genes were identified with cut-offs of *p*-value < 0.05 and fold change > 1.5. Less differentially expressed genes were identified by DEseq2 than edgeR, and results from DEseq2 were applied for follow up analysis to ensure robustness. Gene expression similarity was indicated using Spearman’s correlation coefficient based on gene expression fold change. GO enrichment analysis was performed using clusterProfiler (R version 4.4.1), and pathway analysis was performed using Ingenuity Pathway Analysis (IPA, Qiagen, Hilden, Germany).

### 4.3. UVA-Challenged 3D Full Thickness Skin Model and Histological Analysis

3D full thickness skin models (Fulkutis^®^) were purchased from Guangdong Biocell Biotechnology (Dongguan, China). For experiments examining the dose-dependent effects of RE on collagen and inflammation, the models were irradiated with UVA at 35 J/cm^2^, once per day, for 4 days. RE was applied topically on day 1 and 3 after UV exposure.

For experiments on inflammaging, the models were irradiated with UVA at 10 J/cm^2^, once per day, for 7 days. RE was applied topically on day 1, 3, 5, and 7 after UV exposure. 0.5 ng/mL IL-1β (Sangon Biotech, Shanghai, China) and 0.5 ng/mL TNF-α (MedChemExpress, Monmouth Junction, NJ, USA) were added in the culture medium.

Twenty-four hours after the last UV irradiation, all models (half for histological analyses and immuno-staining, half for RNA extraction) and culture media were collected for further analysis. H&E staining was conducted for histological analysis. The models were immediately fixed in 4% paraformaldehyde for 24 h, then processed and embedded in paraffin. Tissues were sliced into 5 μm sections using a microtome. Sections were further deparaffinized, rehydrated through graded ethanol series, and stained with H&E before being cover slipped. Then the slides were examined under a microscope (Olympus, BX53, Center Valley, PA, USA) and photographed. The boundaries of the living cell in the epidermal layer were drawn, and the average distance between the boundaries was considered as the epidermis thickness, calculated using Image Pro Plus.

### 4.4. IHC and IF Staining Analyses

Harvested HDF and SH-SY5Y cultures were fixed using 4% paraformaldehyde, rinsed with PBS and then permeabilized. Paraffin-embedded sections from 3D full thickness skin models were deparaffinized and rehydrated. Antigen retrieval was performed using a 0.01 M sodium citrate buffer solution at high pressure. After these steps, cells or sections were washed and blocked, IHC and IF staining were performed according to the manufacturer’s instruction for specific antibodies: Collagen I (Cell Signaling Technology, Danvers, MA, USA, 72026), Collagen IV (Abcam, Waltham, MA, USA, ab6312), Laminin 332 (Abcam, ab78286), Filaggrin (Abcam, ab218397), and Collagen XVII (Abcam, ab186415).

For IHC staining, the sections were finally incubated with a DAB chromogen and dehydrated before being cover slipped. Then, the slides were examined under a microscope (Olympus, BX53) and photographed. For IF staining, nuclei were stained with Hochest before being cover slipped. Then the slides were examined under a microscope (Olympus, BX43) and photographed. All staining images were quantified with Image Pro Plus in triplicate.

### 4.5. ELISA

Culture supernatants of skin models were collected to analyze levels of IL-1α, IL8, and MMP1, which were measured using the respective ELISA kits (Abcam) following the manufacturer’s instructions.

### 4.6. Reverse Transcription-Quantitative Polymerase Chain Reaction (RT-qPCR) Assay

Total RNA was isolated from cells using RNeasy Mini Kit (Qiagen) according to the manufacturer’s protocol. The quality and quantity of the extracted RNAs were assured by a Nanodrop spectrometer (Thermo Fisher Scientific). Purified RNA was reverse transcribed to generate the template cDNA using the PrimeScript™ RT Master Mix (Takara Bio Inc., Shiga, Japan). Amplification of target genes was performed with TB Green^®^ Premix Ex Taq™ II (Takara Bio Inc.) on the ABI Vii 7 Real-Time PCR Systems (Applied Biosystems, Thermo fisher scientific, Waltham, MA, USA) with corresponding primers: *IL1A*: F: 5′-TGGCGTTTGAGTCAGCAAAG-3′, R: 5′-AGCACACCCAGTAGTCTTGC-3′; *MMP1*: F: 5′-AAGGTGGACCAACAATTTCAGA-3′, R: 5′-TGAAGGTGTAGCTAGGGTACATCAA-3′; *COL1A1*: F: 5′-GTGGCAGTGATGGAAGTGTG-3′, R: 5′-AGGACCAGCGTTACCAACAG-3′; *IL8*: F: 5′-TGTACTCATGACCAGAAAGACC-3′, R: 5′-ACCAAGGCACAGTGGAACAA-3′; *PTGS2*: F: 5′-CCAGCACTTCACGCATCAG-3′, R: 5′-CATCAGACCAGGCACCAGAC-3′. *18S* was selected as the housekeeping gene. The fold changes in gene expression were calculated with 2^−∆∆Ct^ relative to the non-treated (NT) group.

For skin models, samples were lysed with RNAex Pro Reagent (Accurate Biotechnology, AG21101, Changsha, China) and total RNA was extracted with chloroform. RNA concentration was then measured with a Nanodrop spectrometer (Thermo Fisher Scientific) and RNA was reverse transcribed to cDNA with the Evo M-MLV RT Premix for qPCR (Accurate Biotechnology, Cat: AG11706) according to manufacturer’s instructions. qPCR was performed with SYBR^®^ Green Premix Pro Taq HS qPCR Kit (Accurate Biotechnology, AG11701) on Lightcycler 480 II (Roche, Basel, Switzerland) with corresponding primers: *CDKN2A*: F:5′-GTGGACCTGGCTGAGGAG-3′, R:5′-ATGGTTACTGCCTCTGGTG-3′; *CDKN1A*: F:5′-ATGTCCGTCAGAACCCATGC-3′, R:5′-GCCATTAGCGCATCACAGTC-3′. *ACTB* was selected as the housekeeping gene. The fold changes in gene expression were calculated with 2^−∆∆Ct^ relative to the NT group.

### 4.7. Statistical Analysis

Data were analyzed using GraphPad Prism 10 (GraphPad Software, San Diego, CA, USA). The results of the in vitro tests are presented as mean ± standard deviation (SD). Results were statistically analyzed using Student’s unpaired *t*-test. A *p*-value < 0.05 was considered statistically significant.

## 5. Conclusions

In conclusion, RE represents a promising candidate for the development of new antiaging interventions, particularly for populations sensitive to conventional retinoids. Through mechanisms such as retinol-like gene regulation, enhancing the synthesis of key structural proteins such as collagen, protection against inflammaging, and suppression of neurogenic inflammation, RE offers a comprehensive approach to skin aging. These combined actions result in broader anti-aging benefits, including improvements in skin texture and resilience, while maintaining a favorable safety profile that supports long-term application.

## Data Availability

The original contributions presented in this study are included in the article. Further inquiries can be directed to the corresponding author.

## Abbreviations

The following abbreviations are used in this manuscript:

RE	Rosemary extract
HDF	Human dermal fibroblasts
NC	Negative control
ECM	Extracellular matrix
DEJ	Dermal-epidermal junction
TRPV1	Transient receptor potential vanilloid subtype 1
MMP1	Matrix metalloproteinase 1
IF	Immunofluorescence
IHC	Immunohistochemical
RT-qPCR	Reverse transcription-quantitative polymerase chain reaction
SD	Standard deviation
